# Identifying primary care quality indicators for people with serious mental illness: a systematic review

**DOI:** 10.3399/bjgp17X691721

**Published:** 2017-07-04

**Authors:** Christoph Kronenberg, Tim Doran, Maria Goddard, Tony Kendrick, Simon Gilbody, Ceri R Dare, Lauren Aylott, Rowena Jacobs

**Affiliations:** National Research Center for Health Economics, University of Duisburg-Essen, Essen, Germany.; Department of Health Sciences;; Department of Health Sciences;; Primary Care and Population Sciences, University of Southampton, Southampton, UK.; Department of Health Sciences;; Expert by experience, UK.; Expert by experience, UK.; Centre for Health Economics, University of York, Heslington, York, UK.

**Keywords:** pay-for-performance schemes, primary care, quality indicators, serious mental illness, systematic review

## Abstract

**Background:**

Serious mental illness (SMI) — which comprises long-term conditions such as schizophrenia, bipolar disorder, and other psychoses — has enormous costs for patients and society. In many countries, people with SMI are treated solely in primary care, and have particular needs for physical care.

**Aim:**

The objective of this study was to systematically review the literature to create a list of quality indicators relevant to patients with SMI that could be captured using routine data, and which could be used to monitor or incentivise better-quality primary care.

**Design and setting:**

A systematic literature review, combined with a search of quality indicator databases and guidelines.

**Method:**

The authors assessed whether indicators could be measured from routine data and the quality of the evidence.

**Results:**

Out of 1847 papers and quality indicator databases identified, 27 were included, from which 59 quality indicators were identified, covering six domains. Of the 59 indicators, 52 could be assessed using routine data. The evidence base underpinning these indicators was relatively weak, and was primarily based on expert opinion rather than trial evidence.

**Conclusion:**

With appropriate adaptation for different contexts, and in line with the relative responsibilities of primary and secondary care, use of the quality indicators has the potential to improve care and to improve the physical and mental health of people with SMI. However, before the indicators can be used to monitor or incentivise primary care quality, more robust links need to be established, with improved patient outcomes.

## INTRODUCTION

Serious mental illness (SMI) includes schizophrenia, bipolar disorder, and other psychoses (defined by International Classification of Diseases [ICD-10]^1^ categories F20–F31, and including schizophrenia spectrum and other psychotic disorders together with bipolar and related disorders in DSM-5).^2^ SMI is linked with poor health outcomes, high healthcare costs, and high disease burden.^3^^,^^4^ People with SMI have, on average, a 20-year lower life expectancy, mostly due to preventable causes.^5^^–^8 The global morbidity study attributed 3.5% of total years lost to disability to schizophrenia and bipolar disorder combined.^9^ SMI is also associated with increased treatment costs^10^ and hospitalisations. Yet, around a third of people with SMI in the UK are treated solely in primary care,^11^ and are in long-term contact with primary care services more often than the general population.^12^^,^^13^ Even in countries with very well developed secondary mental health care systems, primary care can make a key contribution to the care of people with SMI.^14^ The quality of primary care for people with mental health problems is therefore of international concern.^15^^,^^16^

In the UK, a national pay-for-performance scheme, the Quality and Outcomes Framework (QOF), exists to financially reward family practices for achieving quality targets for patients with long-term conditions. The SMI quality indicators in the QOF cover both mental health specific care (for example, monitoring lithium levels) and more general physical care (such as routine health checks). QOF indicators are for high-priority disease areas for which primary care has principal responsibility for ongoing care, and where there is good evidence that improved primary care will have health benefits. However, the QOF may neglect important unmeasured aspects of quality of care,^17^ and the incentives may result in tunnel vision,^18^ or a focus on activities that are prioritised at the expense of other non-incentivised activities.^19^^,^^20^ For example, the QOF focuses more on physical than mental health, because this is generally easier to measure.

The authors performed a systematic review of the literature and interrogated international databases to identify potential quality indicators that could supplement or replace indicators already included in the QOF for people with SMI, and which could potentially be incentivised in primary care. The authors included indicators that appeared in earlier versions of the QOF but were subsequently dropped from the scheme when it was reduced in scope to reduce workload. These indicators were included on the grounds that they remain valid measures of quality of care, and continue to be included in the broader National Institute for Health and Care Excellence (NICE) indicators menu. A major focus of the analysis was the source of the data on which the indicators were based. Those requiring primary data collection — for example, via surveys of patients or health professionals, or retrospective auditing of patient records — would be very challenging to incorporate into incentive schemes such as the QOF, whereas those based on routinely available data would, in principle, be more feasible to establish.

**How this fits in**

This is the first systematic review of indicators of primary care quality for patients with serious mental illness (SMI). The study identifies 59 quality indicators in six domains, the majority of which could be monitored using routine primary care data. A key domain is the focus on physical health care. Consideration of the use of a broad set of quality of care indicators may support the improvement of the mental and physical health of this patient group.

Previous literature reviews on quality indicators have focused on SMI in secondary care,^22^^,^^23^ whereas this study (to the authors’ knowledge) is the first to focus specifically on people with SMI in primary care. Identifying indicators of primary care quality for people with SMI could help to strengthen the evidence base and shed light on neglected areas of care, as well as providing the basis for incentive schemes aimed at improving quality.

## METHOD

A systematic review of primary care quality indicators for people with SMI was conducted with the aim of identifying quality indicators in addition to those already included in the QOF, either in the past or currently.

### Inclusion and exclusion criteria

The authors searched for published examples of potential quality indicators that could readily be collected in primary care with reference to routine data. Search terms were identified by an information specialist in conjunction with the project team. Included papers had the terms serious mental illness AND primary care AND quality indicator, including alternative spellings and synonyms. Studies on children or covering non-psychotic illnesses, for example, severe depression or anxiety disorders, were excluded. All studies from January 1990 to February 2015 were considered for inclusion. No language restrictions were applied, although all search terms were in English, and all studies in English, German, Dutch, and Afrikaans were considered due to authors’ language knowledge. The base search was constructed using MEDLINE and adapted to the other resources. The following databases were searched: Applied Social Sciences Index and Abstracts (ASSIA); CENTRAL; Cochrane Database of Systematic Reviews; Conference Proceedings Citation Index-Science (CPCI-S); Database of Abstracts of Reviews of Effects (DARE); EMBASE; Ovid MEDLINE^®^ In-Process & Other Non-Indexed Citations and Ovid MEDLINE^®^; PsycINFO; and MEDLINE. The full strategy for MEDLINE as a template is available in Appendix 1.

Additionally, previous reviews with overlapping aims were searched, and authors were contacted to ask for their indicators (most notably Stegbauer *et al*^22^ and Großimlinghaus *et al*^24^). The quality indicator database of the Agency for Healthcare Research and Quality (AHRQ)^25^ was also searched for indicators relevant to primary care. The final selection of indicators was informed by the views of the study steering committee, which included service users.

### Study selection

Titles of papers were first reviewed by five authors, and all studies that had been identified as potentially relevant by two members of the research team were included in the abstract screening process. All abstracts were screened by six authors, and full papers were obtained if two members of the team judged the abstract potentially relevant or in scope, that is, covering serious mental illness, primary care, and quality indicators. Full papers were divided into four groups and independently reviewed by four pairs of authors. The focus of the selection was to identify papers that included relevant quality indicators that could be applied in primary care. It was evident that the definition of primary care varies between different countries, so the authors included indicators with elements of shared care between primary and specialist settings (for example, prescribing and monitoring of antipsychotic medication), while acknowledging that, in some countries, those indicators may be more applicable to secondary care.

The search strategy complied with the PRISMA checklist (Appendix 2).

### Data extraction and analysis

From each paper, a short description of each indicator was extracted, and the descriptions for similar indicators were merged. After reviewing the general areas covered by the indicators, they were grouped into six domains (coordination of care, substance misuse, service provision and access to care, medicines management, mental health assessment and care, and physical health assessment and care). The domains were selected by the research team, which included service users, as representing broad areas of service provision and care that were viewed as important and could encompass all the chosen indicators. Some of the indicators may overlap the domain description boundaries as they are not intended to be rigid boundaries. Given the main focus of the study, the authors decided whether each indicator could, in principle, be measured from routine data or whether primary data collection would be necessary. Furthermore, the authors checked whether the identified indicators had ever been included in the QOF. They also assessed the quality of the evidence of the included studies using an adaptation of the GRADE guidelines,^52^ and rated the quality of the evidence as high (systematic reviews or randomised control trials), moderate (nonrandomised control studies or unsystematic reviews), low (expert opinion or uncontrolled studies), or not applicable (measure was extracted from the grey literature).

## RESULTS

**Figure 1.**
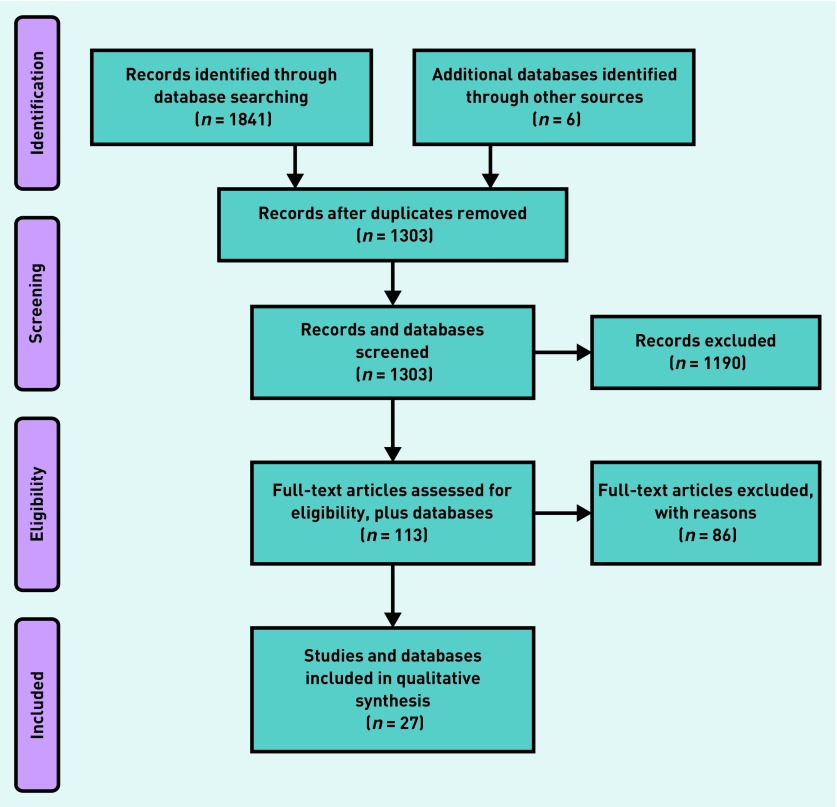
***PRISMA^21^ flow diagram for systematic review of quality of care indicators for patients with serious mental illness.***

In total, 1847 studies and further database sources were identified using the search. The split was ASSIA (34), CENTRAL (96), Cochrane (12), Conferences Proceedings (125), DARE (28), EMBASE (738), Ovid MEDLINE (537), PsycINFO (271), and six further database sources (AHRQ,^25^ Stegbauer *et al*,^22^ Großimlinghaus *et al*,^24^ Parameswaran *et al*,^23^ and NICE).^30^^,^^37^ After removing duplicates using bibliographic software (EndNote and Zotero), 1303 records remained. Title screening reduced this to 356, excluding those that were not about quality indicators, or primary care, or mental illness, or were not included in the definition of SMI (for example, depression or substance misuse disorders). Abstract screening reduced the records to 113, with similar reasons for exclusion. Finally, from those 113 records, 86 were excluded, and 27 records were included in the review (Figure 1, PRISMA flow diagram). From these 27 records, a final set of 59 different indicators was extracted.

Table 1 shows the final list of indicators. Of the 59 indicators, 52 could potentially be assessed using routine data, and seven would require primary data collection from patients or professionals. Of these 59 indicators, 17 are, or have previously been, included in the QOF. A large proportion of the indicators relevant to primary care are in the physical health domain. Another large subset of indicators relate to the process of receiving care, for example, continuity of care, access to services, and frequency of contacts.

**Table 1.** Quality of care indicators identified for people with serious mental illness

**Number**	**Description**	**Data source**	**QOF**	**AHRQ**	**References**
**Coordination of care**					
1	Coordinated care — identify key worker (social worker or CPN)	Routine data			26
2	Staff continuity — good communication between staff and infrequent staff changes	Routine data			27
3	Continuity: CONNECT is a patient questionnaire with 72 items, each rated on a five-point scale, with 13 scales and one single-item indicator: General coordination — ‘Overall, is your mental health treatment well coordinated?’ Primary care scales — ‘How often is psychiatrist in contact with your primary care doctor?’ (Never, Rarely, Sometimes, Often, Always)	Primary data			28
4	Total number of follow-up contacts during treatment episode after initial evaluation	Routine data			29
**Substance misuse**					
5	Patients with SMI who smoke who are offered tobacco counselling/help to stop smoking	Routine data			25^,^^30^
6	Alcohol misuse screening	Routine data	**✓**	**✓**	25
7	Screening for illicit drug use, type, quantity, and frequency	Routine data		**✓**	25
8	Referral to substance misuse disorder specialty care, if appropriate	Routine data			23
9	HIV screening with co-occurring substance misuse for SMI service users	Routine data			31
**Service provision and access to care**					
10	Practice can produce register of all SMI patients	Routine data	**✓**		32
11	Service user registration with a primary health organisation	Routine data			23
12	Markers of care recorded: contact with secondary health services, written care plans, 6-month mental health review, identified care coordinator, evidence of physical examination	Routine data	**✓**		33
13	Patients who do not attend the practice for their annual review who are identified and followed up by the practice team	Routine data	**✓**	**✓**	25
14	System contact: number of patients in contact with the treatment system	Routine data			23
15	Surveillance to prevent relapse	Routine data			27
16	Crisis management and out-of-hours services	Routine data			28
17	Access to services and range of services	Routine data			27
18	Family care — record of families living with person with schizophrenia	Primary data			26
19	Duration of untreated psychosis: number of recently diagnosed patients	Routine data			23^,^^34^
20	Waiting time between registration and start of treatment	Routine data			23
**Medicines management**					
21	All current medication clearly available at all consultations — known drug dosages, frequencies, history of side effects, review date	Primary data			26
22	Monitor patients suffering extra pyramidal effects, check compliance	Routine data			35
23	Assess weight gain, use of concomitant medication	Routine data	**✓**		36
24	Use of lithium: plasma lithium levels monitored regularly	Routine data	**✓**		37^,^^38^
25	Percentages of bipolar service users prescribed antidepressants and anxiolytics	Routine data			37^,^^38^
26	Proportion of patients who are receiving depot antipsychotics who have appropriate laboratory screening tests	Routine data	**✓**		25
27	Patients have their antipsychotic medication reviewed regularly, considering symptoms and side effects: appropriate referral to specialist	Routine data			39^,^^40^
28	Polypharmacy: reduce number of patients using more than four psychotropic drugs at the same time	Routine data			41
29	Monitoring patients with neurological, sexual, sleeping, and sedation side effects	Routine data			42
**Mental health assessment and care**					
30	Percentage of patients given annual mental health review by GP	Routine data	**✓**		43
31	Comprehensive mental status examination and history conducted in patients with a new treatment episode	Routine data	**✓**		25^,^^26^
32	Referral for specialist mental health assessment	Routine data			37
33	Comprehensive assessment of comorbid psychiatric conditions and response to treatment	Routine data		**✓**	25
34	Reassess severity of symptoms	Routine data	**✓**		44
35	Examined for duration of untreated psychosis	Primary data			34
36	Delayed diagnosis	Primary data			45
37	Informal carer contacts	Primary data			27
38	Information on employment status	Primary data			26
**Physical health assessment and care**					
39	Diabetes monitoring for people with diabetes and schizophrenia	Routine data		**✓**	25
40	Diabetes and cholesterol monitoring for people with schizophrenia and diabetes	Routine data		**✓**	25
41	Diabetes screening for people who are using antipsychotic medications	Routine data		**✓**	25
42	Blood pressure screening for patients with diabetes	Routine data	**✓**	**✓**	25^,^^46^^–^49
43	Weight management/BMI monitoring	Routine data	**✓**	**✓**	25^,^^46^^–^49
44	Proportion with increased BMI/abdominal waistline	Routine data	**✓**	**✓**	25^,^^46^^–^49
45	Patients with diabetes who received education about diabetes, nutrition, cooking, physical activity, or exercise	Routine data		**✓**	25
46	Counselling on physical activity and/or nutrition for those with documented elevated BMI	Routine data	**✓**	**✓**	25
47	Retinal exam for patients with SMI who have diabetes	Routine data		**✓**	25
48	Foot exam for patients with SMI who have diabetes	Routine data		**✓**	25
49	Hypertension counselling: patients with hypertension who received education services related to hypertension, nutrition, cooking, physical activity, or exercise	Routine data		**✓**	25
50	Hypertension: recording and monitoring patients with hypertension and high blood cholesterol (LDL)	Routine data	**✓**	**✓**	25^,^^46^^–^49
51	Breast cancer screening for women	Routine data		**✓**	25
52	Colorectal cancer screening	Routine data		**✓**	25
53	Proportion of patients who have an increased blood pressure	Routine data	**✓**	**✓**	25^,^^46^^–^49
54	Proportion of patients who have an increased blood glucose level	Routine data	**✓**	**✓**	25
55	Proportion of patients who have low levels of glycosylated haemoglobin	Routine data	**✓**	**✓**	25
56	Proportion of patients who have increased level of blood lipids	Routine data			22
57	Comprehensive physical health assessment with appropriate advice	Routine data	**✓**		44
58	Patients with diabetes who received psychoeducation related to weight (BMI), diabetes (blood glucose levels)	Routine data			50
59	Medical attention for nephropathy	Routine data			51

AHRQ = Agency for Healthcare Research and Quality. BMI = body mass index. CPN = community psychiatric nurse. LDL = low-density lipoprotein. QOF = Quality and Outcomes Framework. SMI = serious mental illness.

Table 2 shows the quality of evidence of the included studies from which the indicators were drawn. Two studies were rated as high quality (Cochrane or systematic review, randomised control trial); three as moderate (non-randomised study or unsystematic review); 19 as low quality (expert opinion, uncontrolled studies); and three were of uncertain quality, having been identified from the ‘grey’ literature (for example, (non-)government organisations’ documents or databases).

**Table 2.** Quality of evidence of studies identifying quality of care indicators for people with serious mental illness

**Study**	**Description of study**	**Strength of evidence^a^**
Parameswaran, Spaeth-Rublee, Pincus^23^	656 measures of quality of mental health care identified in earlier work are rated in importance, validity, and feasibility, using a modified Delphi process	3
NICE^37^	NICE treatment guidelines for bipolar disorder	4
NICE^30^	NICE treatment guidelines for schizophrenia	4
AHRQ^25^	AHRQ provides a database of quality indicators that was used during the grey literature search	4
Lester, Tritter, Sorohan^32^	Focus groups with patients, GPs, and nurses were conducted to explore how to improve care in cases of acute mental health crises	3
Sweeney, Rose, Clement, *et al*^27^	Structured interviews were conducted with 167 individuals suffering from psychoses to establish a concept of service user-defined continuity of care	3
Ware, Dickey, Tugenberg, McHorney^28^	This study reports on the field testing of an interview-based measure of continuity of care	3
Cerimele, Chan, Chwastiak, *et al* 29	Narrative description of 740 primary care patients with bipolar who participated in an MHIP. Quality of care outcomes were derived from patient disease registry	3
Pincus, Spaeth-Rublee, Watkins^44^	Discussion on the barriers to measuring quality of care in the mental health arena, combined with a short list of potential quality measures	3
Holden^26^	This study audited 16 GPs on their care for 266 patients with schizophrenia and observed that the audit led to improved recording of a range of quality indicators	3
Swartz, MacGregor^31^	The authors of this paper argue that in South Africa the role of mental health nurses has been altered to focus on violence, substance misuse, and HIV/AIDS, and should be refocused on psychiatry care in the primary care setting	3
Ruud^34^	The author summarises the literature on quality of care in mental health services in Norway in 2008–2009	3
Highet, McNair, Thompson, *et al* 45	Interviews with 49 patients with bipolar to describe experience in primary care in Australia. Eight themes for improvement of the primary care experience are outlined	3
Lader^35^	Expert review of the standards of care in schizophrenia to reduce side effects while achieving best treatment outcomes	3
Haro, Salvador-Carulla^36^	Observational study following 11 000 patients who were on or changing antipsychotic medication to determine the best course of treatment with respect to symptoms, quality of life, social functioning, and other outcomes	2
Caughey, Kalish Ellett, Wong^38^	Development, expert review, and assessment of the evidence base for, and validity of, medication-related indicators of potentially preventable hospitalisations	3
Busch, Lehman, Goldman, Frank^39^	Observational study examining trends in four measures of quality over time in the US	2
Young, Sullivan, Burnam, Brook^40^	Uncontrolled study looking at differences in quality of care as variations from national guidelines	3
Nayrouz, Ploumaki, Farooq, *et al* 41	Evaluation of an integrated care approach between primary care and community care, focused on patients with SMI	3
McCullagh, Morley, Dodwell^33^	This observational study looks at urban versus rural differences in quality of care for psychoses, as well as the difference in quality of care conditional on contacts with secondary care	3
Rodgers, Black, Stobbart, Foster^43^	Audit of quality of care in 822 Scottish patients with schizophrenia	3
Osborn, Nazareth, Wright, King^46^	Randomised trial to evaluate the impact of a nurse-led treatment to improve screening for CVD in the SMI population	1
Yeomans, Dale, Beedle^47^	Evaluation of a computer-based physical health screening template versus NICE guidelines for the SMI population	3
Mitchell, Delaffon, Lord^48^	A systematic review and meta-analysis of screening practices with respect to metabolic risks for patients with psychosis	1
Roberts, Roalfe, Wilson, Lester^49^	A retrospective view of case notes in 22 GP practices to determine whether patients with schizophrenia receive equitable physical health care	3
Mainz, Hansen, Palshof, Bartels^42^	Description of the Danish National Indicator Project, which intends to document and advance quality of care	3
Druss, Zhao, Cummings, *et al* 51	The study compared diabetes performance measures in US Medicaid enrolees with and without mental comorbidity	2

a Quality of evidence^51^ is categorised as: 1. High — Cochrane or systematic review, randomised control trial. 2. Moderate — non-randomised control study or unsystematic review. 3. Low — expert opinion, uncontrolled studies. 4. Not applicable — measure was extracted from grey literature, for example, (non-)government organisations’ documents or databases. AHRQ = Agency for Healthcare Research and Quality. CVD = cardiovascular disease. MHIP = mental health integration programme. NICE = National Institute for Health and Care Excellence. SMI = Serious mental illness.

Only a very few randomised control trials (RCTs) have evaluated quality indicators. Two RCTs were reviewed in Cimo *et al*,^50^ producing evidence on the effectiveness of lifestyle interventions for people with type 2 diabetes and schizophrenia or schizoaffective disorder. However, more often, indicators were based on expert consensus or small cross-sectional studies.

Many of the indicators identified were derived from a database of indicators produced by the US Agency for Healthcare Research and Quality (AHRQ),^25^ and the strength of evidence underpinning the individual indicators is variable.

## DISCUSSION

### Summary

To the authors’ knowledge, this is the first attempt to identify in a systematic way potential indicators of quality of primary care for people with SMI. Although the authors identify over 50 indicators that could potentially be captured and monitored using routine data, crucially, they note that the quality of the available evidence underpinning the indicators is relatively weak.

### Strengths and limitations

The feasibility of collecting data for any set of quality indicators will vary across different healthcare systems. Many countries have insurance or other systems, which routinely collect activity data in primary care. Some indicators are likely to require more effort to collect (for example, patient questionnaires for perceived continuity of care), and in many cases even routine data collection can prove very challenging. This study focused specifically on finding indicators that could be monitored at relatively low cost to the healthcare system.

The list of quality indicators identified in this study is much broader and more encompassing than the current list of indicators contained in the QOF SMI domain. However, some of the criticisms inherent in the use of quality metrics would remain even if indicators from this broader list were adopted. These include: measuring only what can be measured (in routine data) at the expense of other measures that matter, for example, ‘softer’ measures such as the quality of relationships or the quality of communication;^53^ the risk of prioritising some activities at the expense of other non-incentivised activities;^54^^,^^55^ and the wider impacts of financial incentives and excessive measurement on provider motivation and behaviour.^56^ Moreover, there are gaps in the literature and in the indicators identified, meaning that the service user perspective is not well represented. There is also an absence of quality indicators around aspects of the social environment, such as the stability of housing for people with SMI. Although such factors are important, and may well influence health outcomes, the extent to which primary care could influence these factors may be very limited and hence it may not be appropriate to hold primary care practitioners responsible for improving quality in these domains. The authors also acknowledge that there is an extensive literature in related areas of research that will also refer to very similar quality indicators,^57^ but the search terms were designed to focus on the specific area of interest, and screened out studies where the focus was broader. Finally, the search excluded non-published indicators and those written in languages other than those listed earlier.

### Implications for research and practice

In the UK, to be included in the QOF, quality indicators must be supported by NICE evidence-based clinical guideline recommendations or evidence from systematic reviews. This, along with the need to maintain a manageable panel of indicators, explains why the large majority of indicators identified are not currently part of the QOF. The downside of the QOF approach is that recommendations based on expert consensus are not put forward for inclusion, despite the fact that a body of informed experts would support a *prima facie* rationale for including them. In contrast, the combined views of experts and patients underpin best-practice guidance for those commissioning mental health services in the UK, covering many of the domains identified in this review, suggesting scope for a similar approach to be taken with respect to the QOF.^58^ The adoption of indicators based on expert and patient consensus must ultimately be supported by evidence on cost-effectiveness, but this also applies to indicators based on higher levels of evidence.^59^

Donabedian’s^60^ conceptual framework of quality of care suggests indicators can usually be divided into three subcategories: structure, process, and outcome measures. To date, the evidence for apparent process improvements under incentive schemes leading to improved patient outcomes is mixed. The vast majority of indicators included in this review relate to processes of care, and, although aspects of process are highly relevant, especially to patients, it is important to establish whether quality indicators also promote improved health outcomes. If so, there is a case for their inclusion in the QOF and other initiatives aiming to improve the care of people with SMI. For physical conditions, improvements in processes of care in primary care settings have been found to be associated with modest improvements in intermediate outcomes (for example, cholesterol levels)^61^ and quality of life,^62^ but associations with patient outcomes such as emergency hospital admission are weaker.^63^ For serious mental illness, the evidence is much more limited and suggests that higher provider performance on processes may not be associated with better patient outcomes.^17^

Many of the indicators identified in the study relate to aspects of physical care. People with SMI are at higher risk of physical ill health (particularly diabetes, and cardiovascular and respiratory disease), so clearly focusing on these aspects could help reduce the associated excess morbidity and mortality.^64^ People with SMI are vulnerable, with significant needs for care that may be missed or undertreated, leading to years spent with disabling morbidity and premature mortality. Viron *et al* 14 emphasised that, in the US, as elsewhere:

‘As frontline clinicians, primary care providers have the potential to reduce the health disparities experienced by this population.’

Consideration of the use of a broader set of quality indicators, including those focusing on physical care, may therefore be a positive step. Given the increased risk of diabetes, cardiovascular disease, and respiratory disease in this population, ongoing primary care for people with SMI should focus on disease prevention through tackling obesity and smoking. Similarly, a large set of indicators relate to processes of care, including ongoing contact with relevant services. Targeting comprehensive primary care to people with SMI can also play a crucial role in promoting their engagement with appropriate specialised mental and physical healthcare services, helping them to reach their full potential.

## Appendix 1. Annotated search strategy (MEDLINE via OVID SP)

**Table:**

1 serious mental illness*.tw. (2037)	39 Ambulatory Care/ (36401)
2 serious mental disorder*.tw. (260)	40 or/32–39 (268786)
3 serious psychiatric illness*.tw. (61)	**Line 40 captures terms for primary care**
4 serious psychiatric ill-health*.tw. (0)	41 Quality Indicators, Health Care/ (10737)
5 serious mental ill-health*.tw. (0)	42 (quality adj2 indicat*).tw. (6747)
6 serious psychiatric disorder*.tw. (130)	43 (quality adj2 measure*).tw. (12491)
7 severe mental illness*.tw. (2679)	44 (quality adj2 criteria).tw. (3829)
8 severe mental disorder*.tw. (720)	45 (performance adj2 indicat*).tw. (4837)
9 severe mental ill-health*.tw. (2)	46 (performance adj2 measure*).tw. (14194)
10 severe psychiatric illness*.tw. (128)	47 (performance adj2 criteria).tw. (1367)
11 severe psychiatric disorder*.tw. (379)	48 (incentive* adj3 scheme*).tw. (207)
12 severe psychiatric ill-health*.tw. (0)	49 (incentive* adj3 assess*).tw. (96)
13 major mental disorder*.tw. (288)	50 (incentive* adj3 measure*).tw. (152)
14 major mental illness*.tw. (350)	51 (incentive* adj3 outcome*).tw. (96)
15 major psychiatric illness*.tw. (151)	52 “Standard of Care”/ (1049)
16 major psychiatric ill-health*.tw. (0)	53 (standard* adj2 care).tw. (25676)
17 major psychiatric disorder*.tw. (730)	54 (standard* adj2 healthcare).tw. (400)
18 major mental ill-health*.tw. (0)	55 “Quality of Health Care”/ (58460)
19 schizophrenia/ or schizophrenia, catatonic/ or schizophrenia, disorganized/ or schizophrenia, paranoid/ or shared paranoid disorder/ (86432)	56 (quality adj2 (healthcare or care)).tw. (39007)
20 (Schizophrenia* or schizophrenic or dementia praecox).tw. (90771)	57 patient outcome assessment/ (934)
21 Schizotypal Personality Disorder/ (2217)	58 (patient adj2 outcome assessment*).tw. (70)
22 (disorder* adj2 schizotypal).tw. (702)	59 (patient adj2 outcome measure*).tw. (2492)
23 (disorder* adj1 delusional).tw. (703)	60 proms.tw. (263)
24 Psychotic Disorders/ (32708)	61 patient satisfaction/ or patient preference/ (63756)
25 ((psychotic adj2 disorder*) or (schizoaffective adj2 disorder*) or psychoses or psychosis or schizophreniform).tw. (38127)	62 (patient* adj2 satisfaction).tw. (26024)
26 bipolar disorder/ or cyclothymic disorder/ (32171)	63 (patient* adj2 experience*).tw. (59692)
27 (Bipolar adj2 (disorder* or depression or depressive or psychosis or psychoses)).tw. (22038)	64 (patient* adj2 preference*).tw. (8103)
28 (Manic state* or mania).tw. (8053)	65 quality.tw. (594390)
29 (Manic adj2 (disorder* or depression or depressive or psychosis or psychoses)).tw. (4445)	66 or/41–65 (782974)
30 (cyclothymic disorder* or cyclothymic personalities or cyclothymic personality).tw. (95)	**Line 66 captures terms for quality indicators**
31 or/1–30 (179930)	67 31 and 40 and 66 (551)
**Line 31 captures terms for serious mental illness**	**Line 67 identifies records that contain at least one term for serious mental illness, and at least one term for primary care and at least one term for quality indicators**
32 exp Primary Health Care/ (82203)	68 limit 67 to yr=”1990 -Current” (537)
33 general practitioners/ or physicians, family/ or physicians, primary care/(18403)	**Line 68 applies the date limit**
34 general practice/ or family practice/ (64455)	
35 (family adj2 pract*).tw. (11764)	
36 (primary adj2 care).tw. (89376)	
37 (general adj2 pract*).tw. (69034)	
38 (family adj2 physician*).tw. (12969)	

## Appendix 2. PRISMA checklist^21^ for systematic review of quality of care indicators for patients with serious mental illness

**Table:**

**Section/topic**	**#**	**Checklist item**	**Reported on page #**
**TITLE**			
Title	1	Identify the report as a systematic review, meta-analysis, or both.	1
ABSTRACT			
Structured summary	2	Provide a structured summary including, as applicable: background; objectives; data sources; study eligibility criteria, participants, and interventions; study appraisal and synthesis methods; results; limitations; conclusions and implications of key findings; systematic review registration number.	1
**INTRODUCTION**			
Rationale	3	Describe the rationale for the review in the context of what is already known.	1
Objectives	4	Provide an explicit statement of questions being addressed with reference to participants, interventions, comparisons, outcomes, and study design (PICOS).	N/A
**METHODS**			
Protocol and registration	5	Indicate if a review protocol exists, if and where it can be accessed (for example, web address), and, if available, provide registration information including registration number.	N/A
Eligibility criteria	6	Specify study characteristics (for example, PICOS, length of follow-up) and report characteristics (for example, years considered, language, publication status) used as criteria for eligibility, giving rationale.	2
Information sources	7	Describe all information sources (for example, databases with dates of coverage, contact with study authors to identify additional studies) in the search and date last searched.	2
Search	8	Present full electronic search strategy for at least one database, including any limits used, such that it could be repeated.	11
Study selection	9	State the process for selecting studies (that is, screening, eligibility, included in systematic review, and, if applicable, included in the meta-analysis).	4
Data collection process	10	Describe method of data extraction from reports (for example, piloted forms, independently, in duplicate) and any processes for obtaining and confirming data from investigators.	4
Data items	11	List and define all variables for which data were sought (for example, PICOS, funding sources) and any assumptions and simplifications made.	5
Risk of bias in individual studies	12	Describe methods used for assessing risk of bias of individual studies (including specification of whether this was done at the study or outcome level), and how this information is to be used in any data synthesis.	N/A
Summary measures	13	State the principal summary measures (for example, risk ratio, difference in means).	N/A
Synthesis of results	14	Describe the methods of handling data and combining results of studies, if done, including measures of consistency (for example, I^2^) for each meta-analysis.	N/A
Risk of bias across studies	15	Specify any assessment of risk of bias that may affect the cumulative evidence (for example, publication bias, selective reporting within studies).	N/A
Additional analyses	16	Describe methods of additional analyses (for example, sensitivity or subgroup analyses, meta-regression), if done, indicating which were pre-specified.	N/A
**RESULTS**			
Study selection	17	Give numbers of studies screened, assessed for eligibility, and included in the review, with reasons for exclusions at each stage, ideally with a flow diagram.	6
Study characteristics	18	For each study, present characteristics for which data were extracted (for example, study size, PICOS, follow-up period) and provide the citations.	5
Risk of bias within studies	19	Present data on risk of bias of each study and, if available, any outcome level assessment (see item 12).	N/A
Results of individual studies	20	For all outcomes considered (benefits or harms), present, for each study: (a) simple summary data for each intervention group; and (b) effect estimates and confidence intervals, ideally with a forest plot.	N/A
Synthesis of results	21	Present results of each meta-analysis done, including confidence intervals and measures of consistency.	N/A
Risk of bias across studies	22	Present results of any assessment of risk of bias across studies (see Item 15).	N/A
Additional analysis	23	Give results of additional analyses, if done (for example, sensitivity or subgroup analyses, meta-regression [see Item 16]).	N/A
**DISCUSSION**			
Summary of evidence	24	Summarise the main findings including the strength of evidence for each main outcome; consider their relevance to key groups (for example, healthcare providers, users, and policymakers).	7
Limitations	25	Discuss limitations at study and outcome level (for example, risk of bias), and at review level (for example, incomplete retrieval of identified research, reporting bias).	7
Conclusions	26	Provide a general interpretation of the results in the context of other evidence, and implications for future research.	7
**FUNDING**			
Funding	27	Describe sources of funding for the systematic review and other support (for example, supply of data); role of funders for the systematic review.	8
